# Warm Forming Characteristics of AA7075: Microstructure Interaction Mechanisms and Constitutive Models

**DOI:** 10.3390/ma19040666

**Published:** 2026-02-09

**Authors:** Jia-Fu Wu, Shi-Bing Chen, Yong-Cheng Lin, Gang Xiao, Dao-Guang He

**Affiliations:** 1 School of Mechanical and Electrical Engineering, Central South University, Changsha 410083, China; jia-fuwu@gyu.edu.cn (J.-F.W.); 213711012@csu.edu.cn (S.-B.C.); 2 School of Mechanical Engineering, Guiyang University, Guiyang 550005, China; 3 State Key Laboratory of Precision Manufacturing for Extreme Service Performance, Changsha 410083, China; 4 Huizhou Jinyuan Intelligent Robot Co., Ltd., Huizhou 516006, China

**Keywords:** 7075 aluminum alloy, warm forming, constitutive modeling, microstructural evolution

## Abstract

The AA7075 holds significant importance in the aerospace field. Understanding its microstructure evolution and constitutive relationships during warm deformation is crucial for optimizing forming processes. To this end, isothermal compression experiments were conducted at different temperatures and strain rates to analyze their flow stress behavior. The microstructure evolution was characterized using electron backscatter diffraction (EBSD) and transmission electron microscopy (TEM). Microstructural analysis confirmed that dynamic recovery constitutes the predominant softening mechanism under warm forming conditions. The results indicate that flow stress is highly sensitive to deformation parameters, decreasing with increasing temperature and rising with increasing strain rate. To accurately describe the flow behavior, two distinct constitutive models were formulated: (1) a phenomenological Hensel–Spittel–Garofalo (HSG) model; (2) a novel hybrid machine-learning model that innovatively integrates the Harris Hawks Optimization (HHO) algorithm with an LSTM model. Both constitutive models demonstrate reasonable predictive accuracy. In comparison, the HHO-LSTM model demonstrated a superior ability to capture complex nonlinear relationships, achieving highly precise predictions of flow stress across the full range of deformation conditions tested in this work. The hybrid machine-learning model proposed in this study provides a highly accurate method for describing and predicting the flow behavior of the AA7075 during warm forming, offering a powerful predictive tool for engineering applications.

## 1. Introduction

The AA7075 is extensively utilized in the production of lightweight, high-strength components owing to its remarkable strength-to-weight ratio [1,2]. However, its inadequate formability at ambient temperature and the issues in preserving the as-formed microstructure and characteristics post-hot working provide considerable obstacles [3]. Consequently, warm forming has been adopted as a primary processing route for manufacturing critical components from this alloy. During warm forming, processing parameters ultimately determine the final component properties by influencing the microstructure evolution [4,5]. The intricate rheological properties and microstructural changes during heated deformation render the precise prediction and control of the process a formidable challenge [5]. Therefore, a profound understanding of the macroscopic and microscopic behaviors of the 7075 alloy under warm forming conditions is of paramount importance for optimizing process parameters and enhancing component performance.

Understanding the mechanisms underlying microstructural variation in AA7075 during warm deformation is crucial for elucidating the fundamental characteristics of its macroscopic mechanical behavior. Research reveals that elevated temperatures facilitate the production, movement, entanglement, and annihilation of dislocations, alongside dynamic recovery (DRV) and dynamic recrystallization (DRX), which are the principal mechanisms influencing the alloy’s rheological properties and microstructural evolution. Research indicates that under elevated temperature conditions, the generation, motion, entanglement, and annihilation of dislocations, along with changes in grains, constitute the primary mechanisms governing the alloy’s flow behavior and microstructure evolution [4,6,7]. With the help of advanced characterization techniques, microstructural features (including grain size, morphology, orientation, dislocation density, and subgrain boundary) can be well revealed. For instance, Liu et al. [6] identified the DRV and DRX as the dominant softening mechanisms in 7055 aluminum alloy by observing its microstructural evolution. These microscale observations provide an experimental foundation for establishing accurate macroscopic constitutive models [8].

The development of accurate constitutive models is pivotal for describing and predicting material flow behavior, serving as a cornerstone for optimizing forming processes and conducting numerical simulations [9,10]. Currently, constitutive models describing material behavior during warm and hot deformation primarily fall into three categories: phenomenological models [11,12,13], artificial neural network (ANN) models [14,15], and physics-based models [16,17]. Phenomenological constitutive models, derived from the empirical fitting of experimental data, are highly regarded for their concise formulations and strong engineering practicality [18,19]. Phenomenological constitutive models often lack a direct correlation between their mathematical parameters and actual microstructural evolution. To address this limitation, physically based constitutive models have been developed [20,21]. Although establishing dislocation density-based physical constitutive models is helpful for gaining an in-depth understanding of the macroscopic and microscopic behaviors of AA7075 under warm forming conditions, physics-based models typically involve numerous parameters. Solving these material parameters faces significant challenges [22,23]. Recently, machine learning has demonstrated powerful potential in handling complex nonlinear relationships [24,25]. Models including Long Short-Term Memory (LSTM) can learn the mapping relationship of true stress and forming parameters from extensive experimental data, thereby achieving high-prediction accuracy [8,26,27]. To further enhance predictive performance and generalization capability, hybrid machine-learning models that integrate optimization algorithms with neural networks have emerged as a new research focus [28,29].

This study focuses on the warm deformation behavior of AA7075. The microstructures under different deformation conditions were characterized to elucidate the underlying softening mechanisms. Then, two types of constitutive models were developed: (1) a phenomenological Hensel–Spittel–Garofalo (HSG) model, and (2) a novel hybrid machine-learning model (HHO-LSTM) that innovatively integrates the Harris Hawks Optimization (HHO) algorithm with an LSTM neural network, aiming to achieve high-precision prediction of flow stress. This work provides a high-accuracy predictive method for the warm forming of AA7075, thereby establishing a solid theoretical foundation for subsequent numerical simulation and engineering applications.

## 2. Materials and Experiments

**Figure 1:**
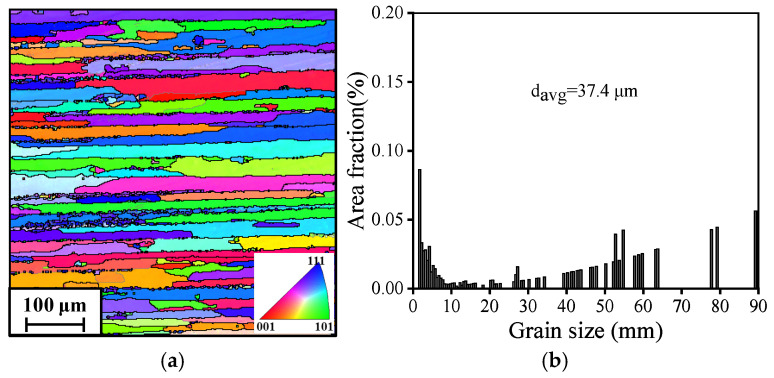
Microstructure of the peak-aged 7075 aluminum alloy: (**a**) inverse pole figure (IPF) map; (**b**) average grain size map. The vertical direction corresponds to the sheet’s thickness direction.

The AA7075 alloy used in this work was in the peak-aged (T6) temper condition. This was achieved by solution treatment at 480 °C for 2 h, water quenching, followed by artificial aging at 120 °C for 24 h. Correspondingly, the chemical composition (wt%) of sheets is 5.7Zn-2.7Mg-1.5Cu-0.21Cr-0.46Fe-0.23Si-0.22Mn-(bal.)Al. The initial microstructure of the sheet was characterized using Electron Backscatter Diffraction (Figure 1). The EBSD analysis reveals that the original material consists primarily of elongated, fully recrystallized grains. A significant proportion of high-angle grain boundaries (marked by black lines in Figure 1b) is observed, with an average grain size of 37.4 μm.

**Figure 2:**
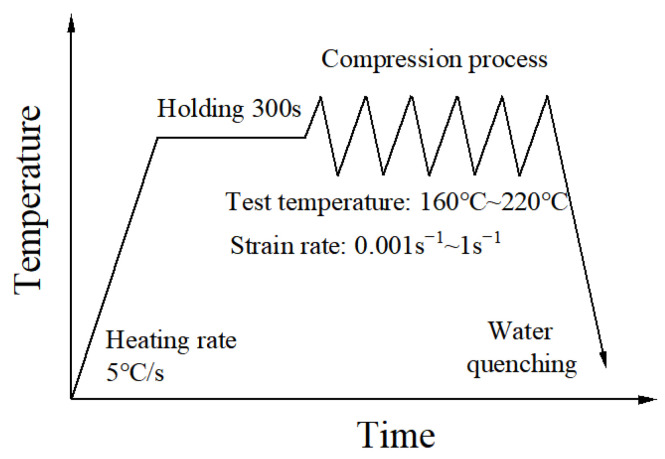
Schematic diagram of the uniaxial warm compression test.

Cylindrical specimens with dimensions of ϕ6 mm×9 mm were machined from the as-received sheets with the cylinder axis parallel to the rolling direction (RD). This orientation was maintained consistently for all compression tests to ensure uniform deformation behavior along the RD. The cylindrical surface was carefully polished with fine sandpaper to eliminate potential stress concentrations caused by surface micro-notches. The two end faces were ground and polished, followed by the application of a graphite-based lubricant to minimize friction during the isothermal uniaxial compression test. The warm compression tests were carried out by the Gleeble thermomechanical processes simulator (DSI, Albany, New York, NY, USA). Four temperature ($T_{w}$) levels (160 °C, 180 °C, 200 °C, 220 °C) and four strain rate ($\dot{\varepsilon }$) levels (0.001 s^−1^, 0.01 s^−1^, 0.1 s^−1^, 1 s^−1^) were employed. During testing, each specimen was heated to the specified temperature at 5 °C/s for 300 s to ensure thermal uniformity. The compression was then carried out under program control according to the preset strain rates. Three replicate experiments were performed under diverse deformation circumstances to assess the results’ consistency. Following deformation, the specimens underwent rapid quenching in water to preserve the high-temperature microstructure for additional analysis. The schematic compression testing procedure is presented in Figure 2.

EBSD analyses were performed on deformed specimens utilizing the NordlysMax2 dual-beam electron microscope to examine the effects of thermal deformation parameters on microstructural evolution. Compression specimens were sectioned along the rolling direction using wire-cut electrical discharge machining (EDM), then underwent grinding and electrochemical polishing to provide specimens appropriate for EBSD analysis. EBSD scans were conducted at an operating voltage of 15 kV, with a magnification of 500× and a scanning area of 640 × 640 μm. Ultimately, EBSD data were examined utilizing HKL Channel 5 software to derive grain size, mean slip angle, and energy distribution metrics. Furthermore, thin sections of 700 μm in thickness were fabricated along the rolling direction. The slices were precision-ground to a thickness of 105 μm and subsequently thinned to the final thickness using a dual-jet thinning system, preparing specimens for transmission electron microscopy (TEM) analysis. The warm-compressed specimens were analyzed with an F20 field emission transmission electron microscope to further explore the development of dislocations and substructures under varying thermal deformation settings.

## 3. Result and Discussion

### 3.1. Examination of Warm Deformation Flow Characteristics

**Figure 3:**
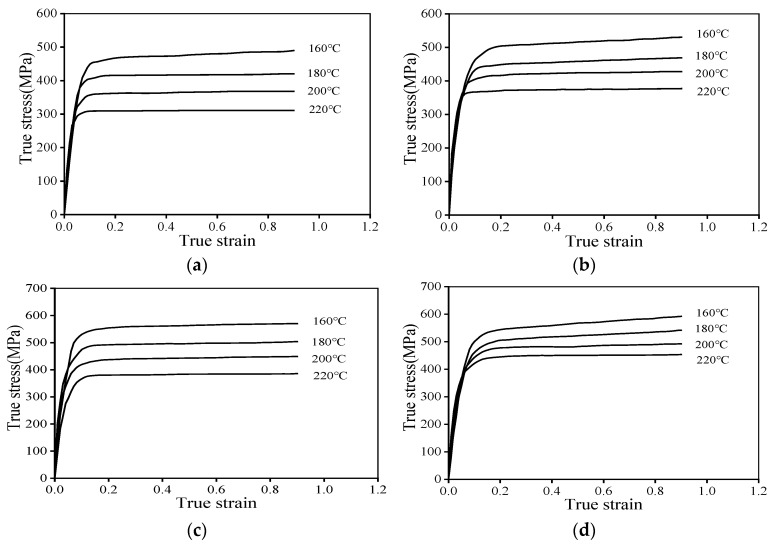
Flow stress curves of the AA7075 during warm deformation at: (**a**) 0.001 s^−1^, (**b**) 0.01 s^−1^, (**c**) 0.1 s^−1^, (**d**) 1 s^−1^.

Figure 3 illustrates the flow stress curves of AA7075 subjected to various thermal deformation conditions. In the preliminary phase of deformation, true stress escalates swiftly with rising true strain, culminating in the achievement of peak stress. This sharp rise is attributed to significant work hardening, where dislocation multiplication occurs swiftly, while dislocation rearrangement and annihilation processes are not yet sufficient. Consequently, a small increment in true strain gives rise to a substantial increase in true stress. DRV is activated once the true strain reaches a critical value, which involves dislocation annihilation and subgrain formation. This process counteracts the dislocation multiplication caused by work hardening. Under specific conditions (i.e., 220 °C and 0.001 s^−1^), the slope of the curve gradually decreases or even approaches zero. However, with further strain increase, no significant stress reduction is observed. This phenomenon arises from aluminum alloys, which possess high stacking fault energy and largely experience dynamic recovery to mitigate the dislocation density produced by work hardening, thereby attaining energy equilibrium. These observations confirm that DRV dominates the softening behavior during the deformation process.

As observed in Figure 3, the warm deformation parameters significantly influence the flow characteristics of the AA7075. Firstly, the flow stress rises with increased $\dot{\varepsilon }$. This trend can be attributed to the longer time available for deformation per unit strain at lower strain rates, which promotes dynamic recovery (DRV) and suppresses dislocation multiplication. Conversely, at higher strain rates, the shorter deformation time restricts DRV, leading to pronounced dislocation accumulation and higher flow stress. Furthermore, Figure 3 indicates that the $T_{w}$ exerting the impact upon flow stress is more pronounced at lower $\dot{\varepsilon }$ (0.001 s^−1^–0.1 s^−1^). Under these lower strain rate conditions, DRV dominates the softening process, resulting in substantial dislocation annihilation. Consequently, variations in temperature induce significant fluctuations in stress. In contrast, at a strain rate of 1 s^−1^, work hardening predominates, where the rate of dislocation multiplication exceeds that of dislocation annihilation. As a result, the effect of temperature on flow stress is relatively less pronounced under high strain rate conditions. The yield stress fluctuations presented in Table 1 align with the preceding analysis. Furthermore, the activation energy for AA7075 aluminum alloy under the current deformation conditions was calculated to be 290.94 kJ/mol, which is consistent with previous literature [30].

### 3.2. Influence of Warm Deformation Parameters on Microstructure

#### 3.2.1. Effect of Deformation Temperature on Microstructure

**Figure 4:**
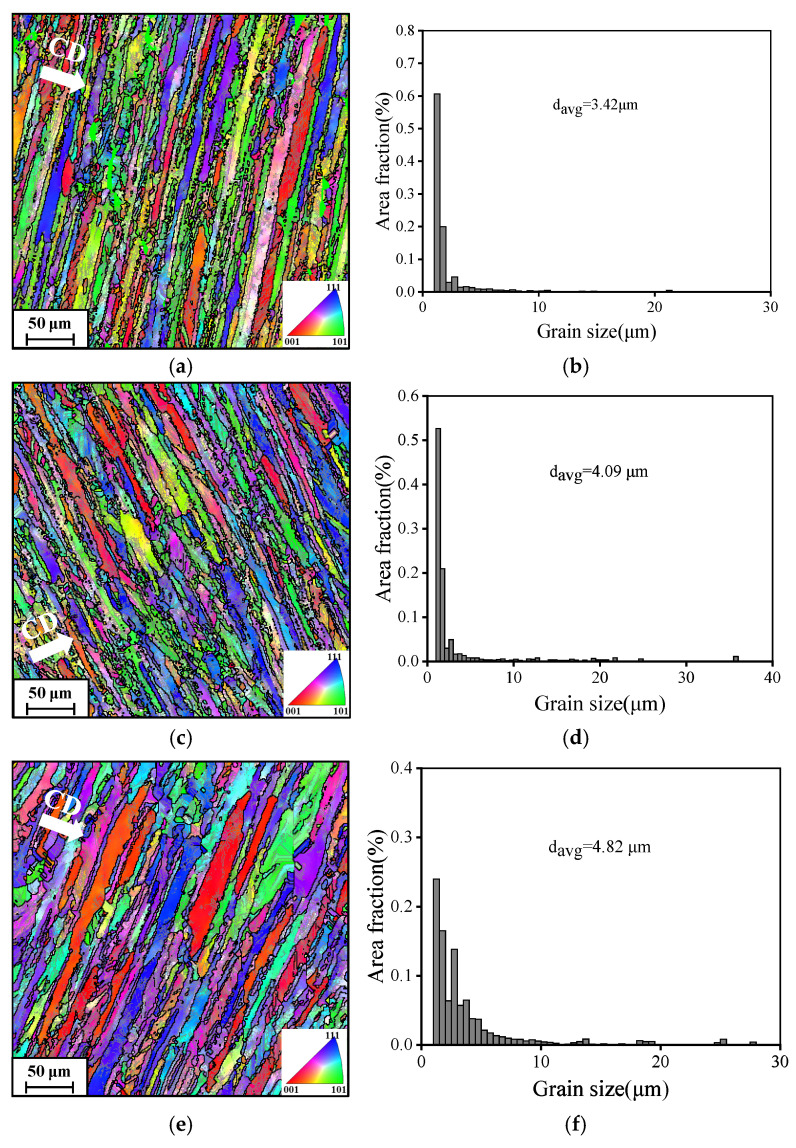
EBSD analysis of specimens compressed at a strain rate of 0.01 s^−1^. (**a**,**c**,**e**) Inverse pole figure (IPF) maps. (**b**,**d**,**f**) Average grain size distribution maps at (**a**,**b**) 180 °C, (**c**,**d**) 200 °C, (**e**,**f**) 220 °C. The symbol ‘CD’ is the compression direction.

Figure 4 depicts the progression of grain shape when the examined alloy undergoes deformation at different temperatures while maintaining a constant strain rate of 0.01 s^−1^. The deformation temperature markedly affects the development of the grain structure. The inverse pole figure (IPF) maps clearly demonstrate that grains deformed by warm compression tend to elongate perpendicular to the compression direction (CD), shown in Figure 4a,c,e. Additionally, substructures are observed along with fine and fragmented grains.

At 180 °C (Figure 4b), the elongated grains contain abundant substructures primarily characterized by low-angle grain boundaries (LAGBs), with a relatively small average grain volume. Statistical analysis indicates an average grain size of 3.42 μm. At 200 °C (Figure 4d), the number of substructures decreases while the average grain volume increases, resulting in an average grain size of 4.09 μm. At $T_{w}$ of 220 °C (Figure 4f), the population of intragranular substructures shows a noticeable decreasing trend, accompanied by a continued expansion in average grain volume and an increased average grain size of 4.82 μm.

As demonstrated above, once the $T_{w}$ raises, the accelerated annihilation in substructures appears. This trend occurs because, at lower temperatures, work hardening is more pronounced, leading to a higher rate of dislocation accumulation and multiplication, thereby generating more substructures. As the temperature rises, DRV is activated and gradually becomes the dominant mechanism, promoting dislocation annihilation and resulting in a reduction in substructure formation. Furthermore, the average grain volume increases with rising deformation temperature. At lower temperatures, significant work hardening facilitates stress concentration, which ultimately leads to grain fragmentation during warm compression, thereby reducing the average grain size. In contrast, at higher temperatures, dynamic recovery plays a more notable role, resulting in more uniform stress distribution and lower overall stress levels. This condition provides a favorable environment for grain growth, resulting in a larger grain size.

**Figure 5:**
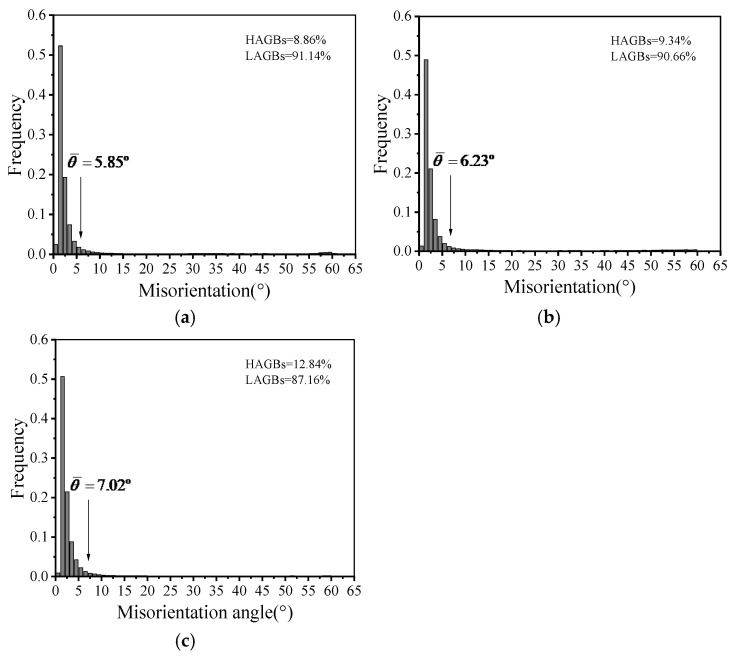
Distributions of grain boundary misorientation angles in specimens compressed at a strain rate of 0.01 s^−1^ and different temperatures: (**a**) 180 °C, (**b**) 200 °C, (**c**) 220 °C.

Figure 5 presents the grain boundary misorientation angle distributions under different compression temperatures, with the relevant data obtained from analysis using the Channel 5 software. Here, LAGBs denote low-angle grain boundaries (0–15°). HAGBs signify high-angle grain boundaries (>15°). The $\bar{\theta }$ represents the average misorientation angle [31]. Under the deformation regime of 0.01 s^−1^ and the temperature range tested, the lower HAGB proportion suggests that softening is primarily accomplished by DRV in the studied AA7075.

Furthermore, statistical results reveal that at temperatures of 180 °C, 200 °C, and 220 °C, the average misorientation angles are 5.85°, 6.23°, and 7.02°, and the fractions of LAGBs are 91.14%, 90.66%, and 80.62%, respectively. The statistics reveal that as the temperature rises, the proportion of LAGBs progressively diminishes. This phenomenon arises because, at a constant strain rate, elevated temperatures facilitate recrystallisation, resulting in the development of new grains that enhance the fraction of HAGBs [32].

**Figure 6:**
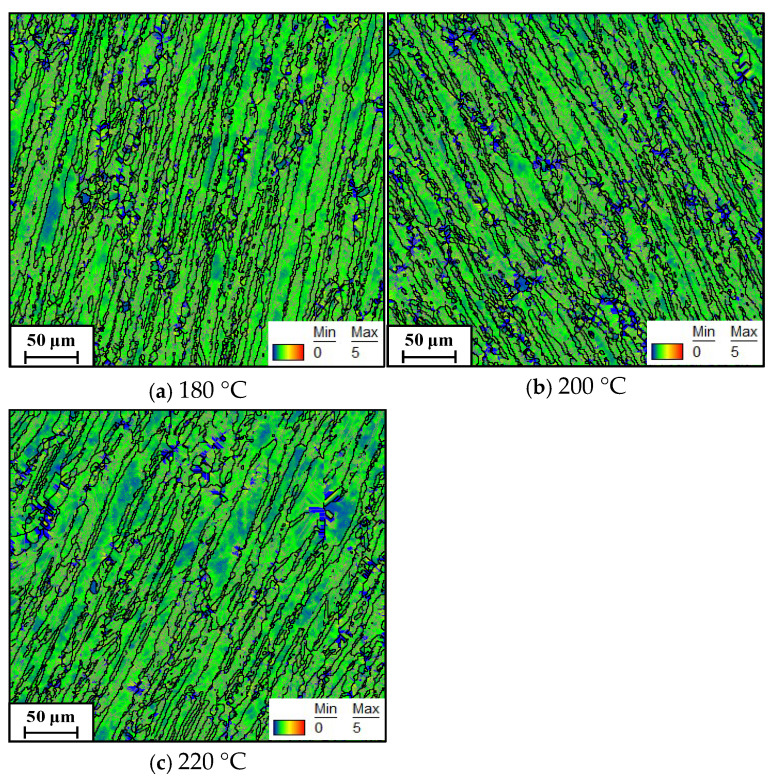
Kernel average misorientation maps showing local strain distribution under a strain rate of 0.01 s^−1^ at different temperatures: (**a**) 180 °C, (**b**) 200 °C, (**c**) 220 °C.

During warm compression deformation, the evolution of LAGBs is associated with the substructures [33]. To gain deeper insight into the variation in LAGBs during warm deformation, the KAM maps were analyzed. Figure 6 presents KAM maps under different compression temperatures at a constant strain rate of 0.01 s^−1^. In these maps, the color transitions from blue to red correspond to an increase in local misorientation angle from 0° to 5°. Higher misorientation angles indicate greater densities of geometrically necessary dislocations (GNDs). As shown in Figure 6a, at 180 °C, a predominance of green-colored areas with limited blue regions is observed, indicating the development of substructures (dislocation networks and subgrains). With increasing deformation temperature (Figure 6b,c), the area covered by blue expands progressively. Statistically, the average KAM values at 180 °C, 200 °C, and 220 °C are 1.67°, 1.58°, and 1.43°, respectively. This decrease in KAM values with rising temperature suggests enhanced annihilation and interaction of dislocations and subgrains. This phenomenon mainly stems from intensified DRV at elevated temperatures, which eliminates a greater number of substructures and dislocations. Additionally, the additional thermal energy at elevated temperatures promotes random jumps of solutes and interstitial atoms, thereby accelerating dislocation migration and annihilation.

Furthermore, it can be observed that blue-colored regions are predominantly distributed along grain boundaries. This occurs because warm compression generates a high density of dislocations, resulting in work hardening. With increasing strain, dislocations migrate and accumulate near grain boundaries. It is important to note that dislocations cannot cross HAGBs due to the discontinuity in Burgers vector between differently oriented grains. Instead, dislocation pile-ups near grain boundaries promote local stress concentration. This concentrated stress may facilitate grain boundary sliding and rotation under applied stress, and can also lead to the nucleation of new dislocations in adjacent grains. These processes contribute to the development of local misorientation gradients, as captured in the KAM maps.

**Figure 7:**
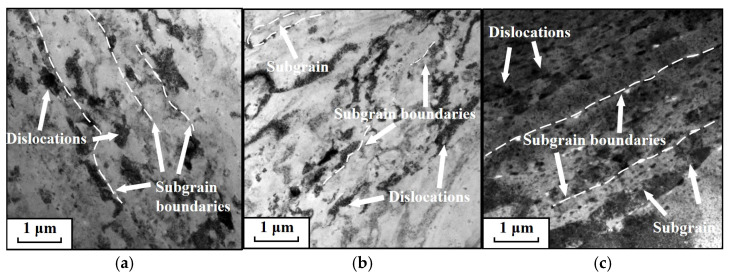
TEM micrographs at a strain rate of 0.01 s^−1^ and: (**a**) 180 °C, (**b**) 200 °C, (**c**) 220 °C. (TEM micrographs taken under two-beam condition with *g* = (111) near the [011] zone axis).

Figure 7 displays TEM micrographs of AA7075 compressed at 0.01 s^−1^ and various temperatures. Evidently, the population and distribution of dislocations, together with substructures, depend on temperature. As shown in Figure 7a, at 180 °C, evidence of severe compressive deformation and accumulation of dislocation subgrains is apparent, with grains being compressed into fine, fragmented morphologies. Dislocations generated by work hardening are distributed along subgrain boundaries, which is consistent with the observations from the KAM analysis. Moreover, with increasing temperature, a noticeable reduction in both dislocation density and the number of subgrains is observed, as illustrated in Figure 7b,c. Most of the remaining dislocations are clustered around subgrain boundaries. This phenomenon is ascribed to the intensified DRV at higher temperatures, which promotes the rapid elimination of substructures and dislocations, resulting in a marked decrease.

#### 3.2.2. Effect of Strain Rate on Microstructure

**Figure 8:**
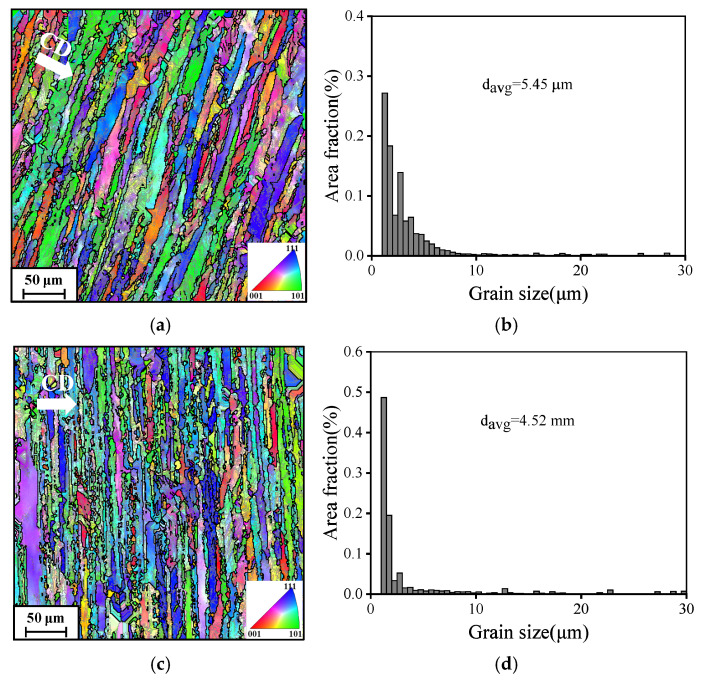
Grain structure and average grain size distribution of the AA7075 under different strain rates at 220 °C: (**a**,**b**) 0.001 s^−1^, (**c**,**d**) 0.1 s^−1^. The symbol ‘CD’ is the compression direction.

Figure 8 illustrates the evolution of grain morphology in AA7075 under different compressive strain rates. Evidently, the strain rate during compression significantly influences the grain structure development. The IPF maps (Figure 8a,c) clearly demonstrate that the deformed grains not only elongate perpendicular to the compression direction but also exhibit a noticeable reduction in grain width and a tendency toward grain fragmentation. Based on the corresponding average grain size distribution maps (Figure 8b,d), the average grain sizes at 0.001 s^−1^ and 0.1 s^−1^ are determined to be 5.45 μm and 4.52 μm, respectively. This phenomenon results from the increased deformation stress exerted on the grains per unit time at elevated strain rates, leading to enhanced grain fragmentation and a subsequent decrease in average grain size [34]. Moreover, the quantity of substructures escalates with rising strain rate. Higher strain rates inhibit dynamic softening mechanisms, resulting in work hardening becoming the predominant process [35]. This, in turn, accelerates dislocation multiplication and accumulation, leading to the formation of more substructures [36].

**Figure 9:**
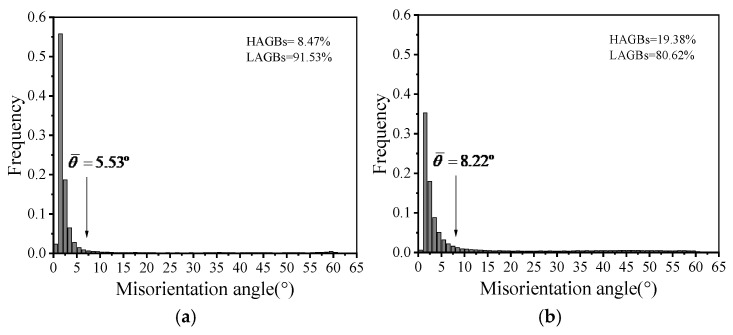
Grain boundary misorientation angle distributions at a deformation temperature of 220 °C and different strain rates: (**a**) 0.001 s^−1^, (**b**) 0.1 s^−1^.

Figure 9 presents the distributions of grain boundary misorientation angles at different strain rates. In Combined Figure 5, at a constant temperature, an increase in strain rate from 0.01 s^−1^ to 0.1 s^−1^ results in a reduction in LAGBs fraction, but the average misorientation angle rises from 5.53° to 8.22°. Conventionally, a higher strain rate is expected to lead to a lower HAGB fraction, which is indicative of the extent of DRX [37]. However, the experimental results obtained in this study contradict this general trend [38,39]. This unexpected outcome is due to DRV being the main dynamic softening mechanism. The temperature range used in this work was set between 160 °C and 220 °C, which is less than the DRX nucleation temperature. Consequently, at this deformation temperature, a lower strain rate promotes DRV to consume dislocations. Therefore, the critically required strain for recrystallization nucleation cannot build up in the deformed grains. In contrast, the higher strain rate imposes a larger incremental strain per unit time, allowing the grains to reach the nucleation threshold sooner and thereby facilitating dynamic recrystallization DRX. As a result, a higher fraction of HAGBs is observed at elevated strain rates.

**Figure 10:**
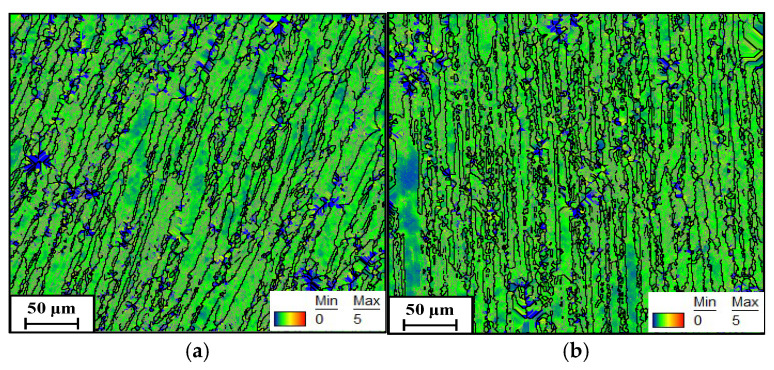
KAM maps showing local strain distribution at: (**a**) 0.001 s^−1^, (**b**) 0.1 s^−1^.

Figure 10 presents the KAM maps of specimens compressed at 220 °C across various strain rates. With increasing strain rate, the blue-colored areas diminish, while green-colored regions become more predominant. Statistically, the average KAM values at 0.01 s^−1^ and 0.1 s^−1^ are 1.33° and 1.65°, respectively. These findings demonstrate that an elevation in strain rate drives the multiplication and interaction of dislocations and subgrains. The underlying mechanism can be explained as follows: the longer duration available per unit strain facilitates the progression of DRV at lower strain rates, thereby promoting dislocation annihilation and resulting in a reduction in GNDs. In contrast, higher strain rates restrict the operation of DRV due to the limited deformation time, resulting in accelerated dislocation accumulation and consequently an elevated KAM value [40].

**Figure 11:**
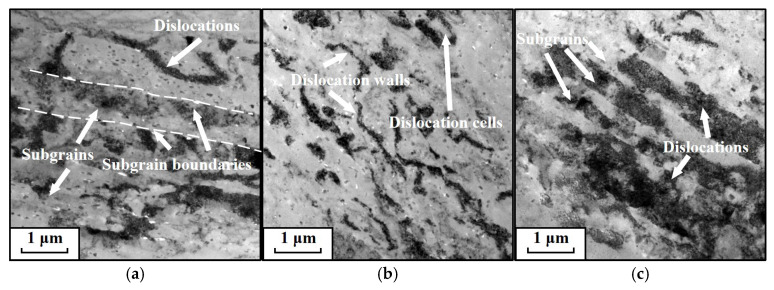
Transmission electron microscopy (TEM) micrographs of specimens deformed at 220 °C under different strain rates: (**a**) 0.001 s^−1^, (**b**) 0.1 s^−1^, (**c**) 1 s^−1^. TEM micrographs taken under two-beam condition with *g* = (111) near the [011] zone axis.

Figure 11 shows TEM images at 220 °C under different strain rates. The observations clearly demonstrate that the population and distribution of dislocations, together with substructures, are strongly dependent on the strain rate. At 0.001 s^−1^ (Figure 11a), numerous subgrains are present, with a relatively low density of dislocations. Under these conditions, the relatively low strain rate provides adequate time for DRV to proceed actively, leading to increased dislocation consumption. At 0.01 s^−1^ (Figure 11b), dislocation cells and dislocation walls formed by extensive dislocation accumulation become clearly visible. The interiors of these dislocation cells are almost free of dislocations. This microstructure develops because, at higher strain rates, dislocations undergo continuous migration and multiplication. When interactions between dislocations themselves and between dislocations and grain boundaries reach a critical level, these dislocations reorganize into cellular structures. At 1 s^−1^ (Figure 11c), the width of the subgrains decreases while their number increases due to the compressive deformation. Simultaneously, the dislocation density multiplies substantially, filling the interior of the grains. This occurs because a very high $\dot{\varepsilon }$ severely shortens the time required for dislocation annihilation through recovery processes, resulting in massive dislocation accumulation.

### 3.3. HSG Model for Flow Behavior of AA7075

Phenomenological constitutive models are commonly employed to characterize the flow behavior of metallic materials [41,42], among which the HSG model serves as a representative example [43,44]:

(1) $$\sinh(\alpha \sigma )=A\exp(k_{1}T)\varepsilon^{k_{2}}{\dot{\varepsilon }}^{k_{3}}\exp(k_{4}/\varepsilon ){\left(1+\varepsilon \right)}^{k_{5}T}\exp(k_{6}\varepsilon )$$

where $k_{1}$, $k_{2}$, $k_{3}$, $k_{4}$, $k_{5}$, $k_{6}$, α and A represent material constants, T, ε and $\dot{\varepsilon }$ represent the deformation temperature, strain, and strain rate, respectively.

Usually, the value in α can be evaluated as follows [45]:

(2) $$\alpha =\frac{{\left(\frac{\partial \ln\sigma_{p}}{\partial \ln\dot{\varepsilon }}\right)}_{T}}{{\left(\frac{\partial \sigma_{P}}{\partial \ln\dot{\varepsilon }}\right)}_{T}}$$

where $\sigma_{P}$ denotes the peak stress.

**Figure 12:**
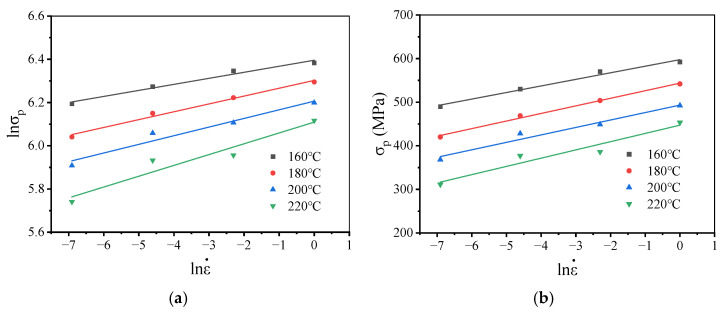
Relationships between: (**a**) $\ln\sigma_{P}$ and $\ln\dot{\varepsilon }$; (**b**) $\sigma_{p}$ and $\ln\dot{\varepsilon }$.

Based on the initial α value from the connections depicted in Figure 12 ($\ln\sigma_{P}-\ln\dot{\varepsilon }$ and $\sigma_{P}-\ln\dot{\varepsilon }$), the value of α for the examined alloy during warm forming was determined to be 0.00892 MPa^−1^ after numerical convergence.

After rearrangement and simplification, Equation (1) is expressed as follows:

(3) $$\ln\sinh(\alpha \sigma )=\ln A+(k_{1}+k_{5}\ln(1+\varepsilon ))T+k_{3}\ln\dot{\varepsilon }+k_{2}\ln\varepsilon +\frac{k_{4}}{\varepsilon }+k_{6}\varepsilon$$

(4) $$k_{3}={\left\{\frac{\partial \ln\sinh(\alpha \sigma )}{\partial \ln\dot{\varepsilon }}\right\}}_{\varepsilon ,T}$$

(5) $$k_{1}+k_{5}\ln(1+\varepsilon )={\left\{\frac{\partial \ln\sinh(\alpha \sigma )}{\partial T}\right\}}_{\varepsilon ,\dot{\varepsilon }}$$

Substituting the value of α into Equations (3)–(5), $k_{1}$, $k_{3}$ and $k_{5}$ can be ascertained. The mean values of A, $k_{1}$, $k_{2}$, $k_{3}$, $k_{4}$, $k_{5}$ and $k_{6}$ are 391,699.03, −0.0181, 0.034, 0.1395, −0.0658,−0.00969 and 2.7361, respectively. Therefore, the HSG model can be expressed as follows:

(6) $$\sinh(0.00892\sigma )=391699.03\exp(-0.0181T)\varepsilon^{0.034}{\dot{\varepsilon }}^{0.1395}\exp(-0.0658/\varepsilon ){\left(1+\varepsilon \right)}^{-0.00969T}\exp(2.7361\varepsilon )$$

**Figure 13:**
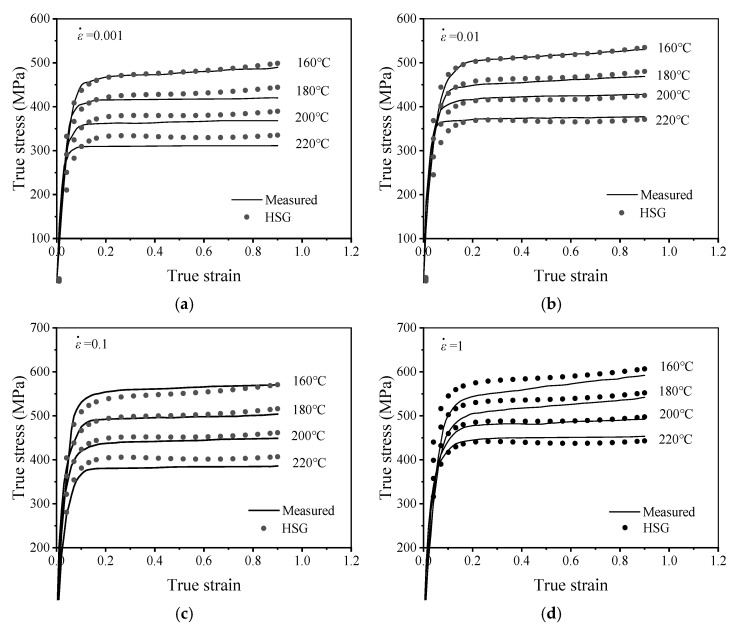
Comparisons between the forecasted tensile stresses by the HSG model and the tested results at: (**a**) $\dot{\varepsilon }$ = 0.001 s^−1^; (**b**) $\dot{\varepsilon }$ = 0.01 s^−1^; (**c**) $\dot{\varepsilon }$ = 0.1 s^−1^; (**d**) $\dot{\varepsilon }$ = 1 s^−1^.

The HSG model was employed to predict the warm tensile characteristics of the AA7075, with the prediction results presented in Figure 13. The curves forecasted by the HSG model exhibit strong concordance with the experimental data, especially at lower $\dot{\varepsilon }$ (0.001 s^−1^, 0.01 s^−1^) and temperatures (160 °C, 180 °C), where the model has significant predictive accuracy. This signifies that the HSG model can characterize the plastic deformation behavior of 7075 high-strength aluminum alloy within this range. At elevated strain rates, the divergence between the predicted and experimental stress values becomes increasingly evident. These phenomena may be ascribed to the more intricate dynamic response, such as adiabatic shear bands [46]. With the rise in $\dot{\varepsilon }$, the alloy has enhanced strain-hardening properties, a phenomenon similarly noted in Reference [47], hence necessitating improved forecast accuracy of the model.

### 3.4. HHO-LSTM for Forecasting Flow Behavior of AA7075

#### 3.4.1. LSTM Model

**Figure 14:**
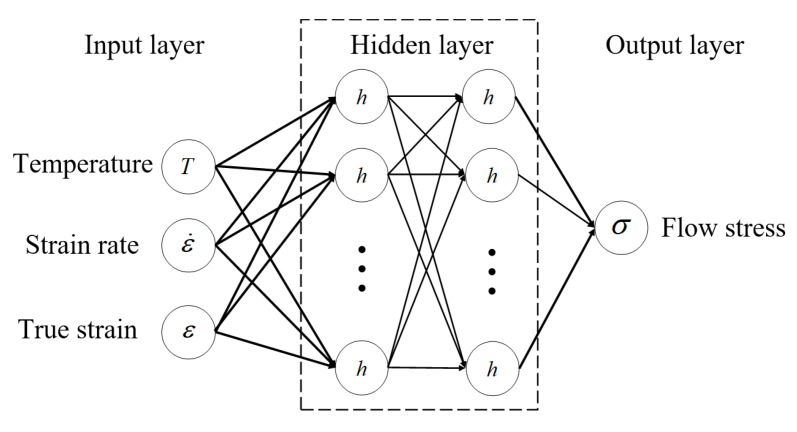
Schematic illustration of the Long Short-Term Memory (LSTM) neural network framework.

The LSTM operates by sequentially and selectively filtering and learning from input data, ultimately training high-precision hyperparameters suitable for the specific data type. Figure 14 illustrates the primary structure of the LSTM model. Given the intricacy of the input data, the number of hidden layers was set to two.

**Figure 15:**
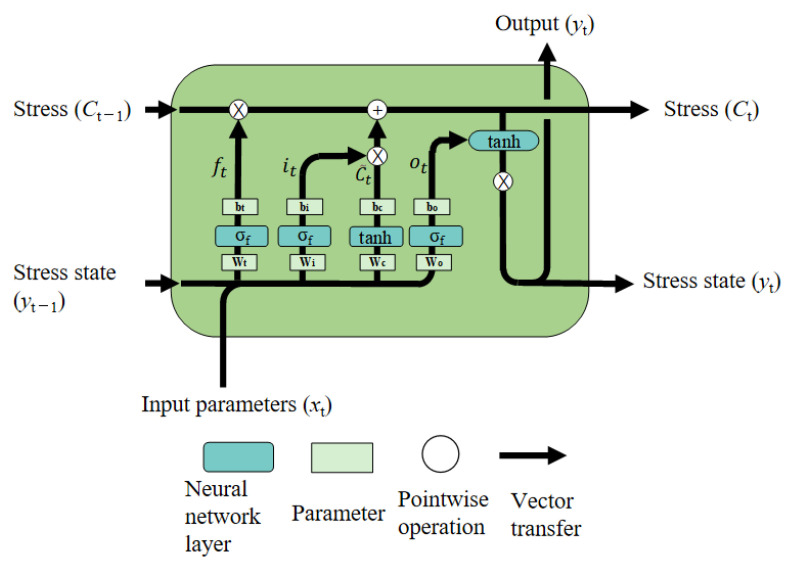
Schematic diagram of the LSTM cell structure and its computational process.

Typically, the data selection and processing in an LSTM model are accomplished through several interconnected “gates”. The collective assembly of these gates, which independently perform data filtering and learning functions, is referred to as a “cell” (Figure 15).

The data filtering and learning processes within an LSTM cell are primarily divided into three components. Here, the $f_{t}$ denotes the forget gate. The $i_{t}$ signifies the input gate. The $o_{t}$ signifies the output gate. The inputs and outputs are denoted as $x_{t}$ and $y_{t}$, respectively. Input data $x_{t}$ and the preceding output stress state $y_{t-1}$ are input into the cell [48].

First, the $f_{t}$ filters out irrelevant information, which is given by the following:

(7) $$f_{t}=\sigma_{f}(W_{f}\cdot [y_{t-1},x_{t}]+b_{f})$$

where $W_{f}$ and $b_{f}$ represent the weight matrix and the bias vector for the forget gate, respectively. $\sigma_{f}$ denote the sigmoid activation function.

The equations for the $i_{t}$ and the stress information (${\tilde{C}}_{t}$) are formulated as follows:

(8) $$i_{t}=\sigma_{f}(W_{i}\cdot [y_{t-1},x_{t}]+b_{i})$$

where $W_{i}$ and $b_{i}$ denote the weight matrix and the bias vector for the input gate, respectively.

(9) $${\tilde{C}}_{t}=\tanh(W_{c}\cdot [y_{t-1},x_{t}]+b_{c})$$

where $W_{c}$ and $b_{c}$ denote the weight matrix and the bias vector for the cell state, respectively.

The $C_{t}$ is given by the following:

(10) $$C_{t}=f_{t}\cdot C_{t-1}+i_{t}\cdot {\tilde{C}}_{t}$$

The data update calculation for the output gate is performed as follows:

(11) $$o_{t}=\sigma_{f}(W_{o}\cdot [y_{t-1},x_{t}]+b_{o})$$

where $W_{o}$ and $b_{o}$ represent the weight matrix and the bias vector for the output gate, respectively.

Finally, by incorporating the activation function, the resulting new hidden state is given by the following:

(12) $$y_{t}=o_{t}\cdot \tanh(C_{t})$$

In Equations (7)–(11), the term [$y_{t-1},x_{i}$] denotes the concatenated input vector.

#### 3.4.2. HHO Algorithm

During this phase, individuals of the hawk population are distributed across different locations within the search area, simultaneously tracking and detecting the prey’s position in the solution space. Global search is performed using two strategies with equal probability. When a random number *I* < 0.5, the hawks update their positions. When *I* ≥ 0.5, the hawks alight randomly on a tree within the confines of the designated area [49]. The mathematical expressions for these two strategies are formulated as follows [50]:

(13) $$H(t+1)=\left\{\begin{array}{ll} \left(H_{prey}(t)-H_{m}(t))-r_{3}(b_{l}+r_{4}(b_{u}-b_{l})\right) & I<0.5\\ H_{r}(t)-r_{1}\left|H_{r}(t)-2r_{2}H(t)\right| & I\geq 0.5 \end{array}\right.$$

where $H_{r}$ represents a random individual in the population at generation ‘t’; $U_{prey}$ denotes the position of the prey; $H_{m}$ represents the average position of the current population; $b_{u}$ and $b_{l}$ indicate the search upper and lower bounds, respectively; and *r*_1_, *r*_2_, *r*_3_, *r*_4_, and *I* are random numbers uniformly distributed in the range (0,1).

The energy for prey’s escape can be calculated as follows:

(14) $$E_{p}=2E_{\mathrm{ini}}(1-\frac{t}{M})$$

where $E_{\mathrm{ini}}$ represents the initial escape energy (from −1 to 1); t and *M* are the current and maximum iteration, respectively. When $\left|E_{p}\right|\geq 1$, the population engages in exploration; otherwise, it switches to exploitation.

The hawk population launches the attack. Actual predatory behavior is highly complex; accordingly, the hawks employ different strategies depending on the situation. Similarly, the HHO algorithm adopts four distinct strategies to capture the optimal solution [49].

When the prey’s escape probability $S_{p}\geq 0.5$. The position update is calculated as follows:

(15) $$H(t+1)=\Delta H(t)-E_{p}\left|JH_{prey}(t)-H(t)\right|$$

When $S_{p}\geq 0.5$ and its escape energy $\left|E_{p}\right|<0.5$, it indicates that the prey lacks sufficient energy for escape and has a limited opportunity for such. The position update is calculated as follows:

(16) $$H(t+1)=H_{prey}(t)-E_{p}\left|\Delta H(t)\right|$$

When $S_{p}<0.5$ and $0.5\leq \left|E_{p}\right|<1$, the position update is calculated as follows:

(17) $$H(t+1)=\left\{\begin{array}{ll} Y=H_{prey}(t)-E\left|JH_{prey}(t)-H(t)\right| & ifF(Y)<F(H(t))\\ Z=Y+S\times {Le}^{\prime }vy(D) & ifF(Z)<F(H(t)) \end{array}\right.$$

When $S_{p}<0.5$ and $\left|E_{p}\right|<0.5$, the prey is exhausted but still has a chance to escape. Consequently, a strategy of high-speed, stepwise stooping is adopted to tighten the siege. The calculation formula is given by Equation (18):

(18) $$H(t+1)=\left\{\begin{array}{ll} Y=H_{prey}(t)-E\left|JH_{prey}(t)-H_{m}(t)\right| & ifF(Y)<F(H(t))\\ Z=Y+S\times {Le}^{\prime }vy(D) & ifF(Z)<F(H(t)) \end{array}\right.$$

#### 3.4.3. Development and Solution of the HHO-LSTM Model

Utilizing the HHO algorithm to fine-tune the hyperparameters of the LSTM model enables the acquisition of a hyperparameter set that yields higher model accuracy and reduced error. The establishment and solution process of the HHO-LSTM model primarily involves three components: data normalization, determination of the algorithm workflow, and model parameter optimization.

**(1)**  **Data normalization**

The LSTM model is constructed with compression temperature, strain rate, and strain as the input neurons, and flow stress as the output neuron. Additionally, the data are standardized using the subsequent equation:

(19) $$d^{\prime }=\frac{d-\bar{d}}{d_{std}}$$

where d signifies the input data, $\bar{d}$ signifies the mean value, and $d_{std}$ signifies the data’s standard deviation.

**(2)**  **Determination of the algorithm workflow**

The LSTM model includes various essential hyperparameters, such as the number of hidden layer nodes, the learning rate, the minimum batch size, and the number of iterations. These four hyperparameters are optimized by the HHO algorithm.

**Figure 16:**
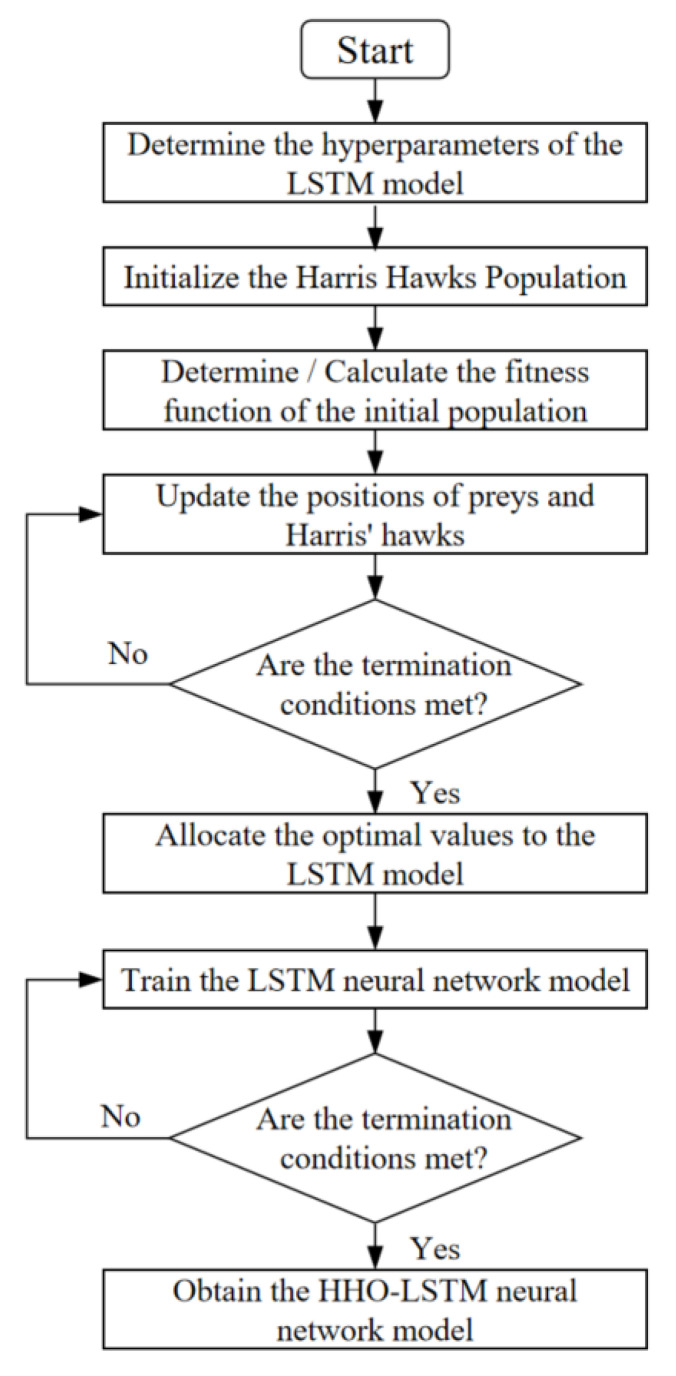
Computational flowchart of the HHO-LSTM model.

The main workflow is illustrated in Figure 16. The four hyperparameters are defined as the optimization targets of the HHO algorithm. The process involves initializing the Harris hawk population, determining and calculating the fitness function of the initial population, and continuously updating the positions of the prey and the hawks. The iteration stops when the convergence criteria are met, and the optimized parameters are then fed into the LSTM model.

**(3)**  **Model parameter optimization**

The warm compression experimental data were arranged into time series, with each deformation condition corresponding to one series, resulting in 16 distinct time series. Subsequently, 80% of the data from all 16 series was fed into the model for training, and the other 20% was retained to validate the model. The HHO-LSTM model computes the mean absolute relative error (*AARE*) between the experimental and predicted values after each evaluation. The iterative process terminates when the correlation coefficient (*R*) reaches 0.99, and the *AARE* is minimized, at which point the optimal hyperparameter values are output. The final optimized hyperparameters obtained via the Harris Hawks Optimization algorithm are as follows: two hidden layers with a total of 100 nodes, the learning rate being set as 0.05, a minimum batch size of 16, and an optimal total iteration count of 1800. These optimized parameters are then input into the model to generate the predicted flow stress data for warm compression.

#### 3.4.4. Validation of the HHO-LSTM Model

**Figure 17:**
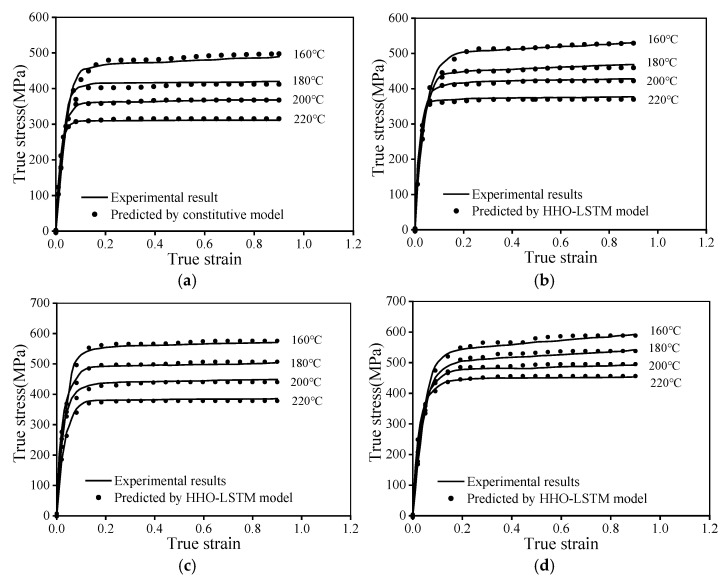
Evaluation of forecasting performance for the HHO-LSTM model at: (**a**) 0.001 s^−1^, (**b**) 0.01 s^−1^, (**c**) 0.1 s^−1^, (**d**) 1 s^−1^.

Figure 17 presents a comparison between the predicted values of the HHO-LSTM model and experimental data, demonstrating the predictive performance of the established neural network model for grasping the macroscopic flow features of AA7075 during warm deformation. Obviously, the HHO-LSTM model exhibits strong agreement with experimental results, accurately capturing the stress–strain behavior of the material at different temperatures. The close alignment between the neural network predictions and experimental warm deformation data can be attributed to the model’s unique “gating unit” computational mechanism, which enables it to precisely capture nonlinear relationships between input and output data.

### 3.5. Verifications of HSG and HHO-LSTM Models

To further assess the model’s predictability, the *R* was used to evaluate the linear correlation between the experimental true stress and the predicted values. The average absolute relative error (*AARE*) was also employed to quantify the prediction error. The *AARE* and *R* values were calculated according to Equation (20) and Equation (21), respectively [51].

(20) $$AARE(\%)=\frac{1}{N}\Sigma_{i=1}^{N}|\frac{X_{i}-Y_{i}}{X_{i}}|$$

(21) $$R=\frac{\sum_{i=1}^{N}\left(X_{i}-\bar{X}\right)\left(Y_{i}-\bar{Y}\right)}{\sqrt{\sum_{i=1}^{N}{\left(X_{i}-\bar{X}\right)}^{2}\sum_{i=1}^{N}{\left(Y_{i}-\bar{Y}\right)}^{2}}}$$

**Figure 18:**
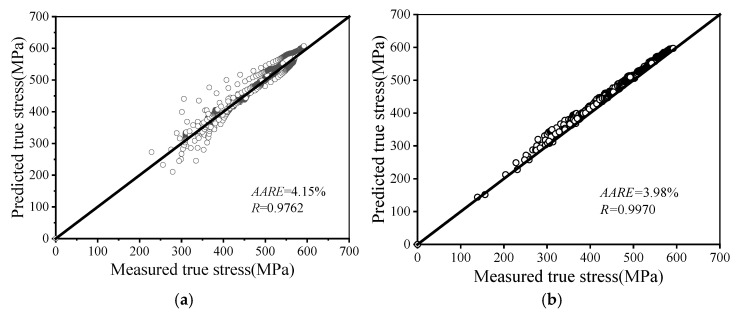
Correlation plot for validating the prediction accuracy of: (**a**) the HSG model; (**b**) the HHO-LSTM model.

Figure 18 presents the comparable forecasting performance of both models. For the HSG model, the *R* and *AARE* are 0.9762 and 4.15%, respectively. In comparison, the HHO-LSTM model achieves an *R* of 0.997 and an *AARE* of 3.98% for its flow stress predictions.

Both models demonstrate strong predictive capabilities with high *R* and relatively low *AARE* values. However, comparative analysis reveals that the HHO-LSTM model exhibits a higher *R* and lower *AARE* value, indicating its superior predictive performance over the HSG model in accurately and efficiently describing the flow behavior of AA7075 under warm deformation conditions.

## 4. Conclusions

This study investigated the warm deformed flow features and microstructure evolution of AA7075, establishing an HSG model and a hybrid HHO-LSTM machine-learning model. These models provide deeper insights into the material’s deformation mechanisms. The main findings are summarized as follows:

(1) The flow stress varying features of AA7075 under warm compression were experimentally determined, showing significant dependence on temperature and strain rate. At higher strain rates or lower deformation temperatures, dynamic recovery (DRV) is suppressed, leading to dominant work hardening, accelerated dislocation multiplication and accumulation, and consequently an increase in flow stress. Conversely, at lower strain rates or higher deformation temperatures, work hardening is inhibited while DRV becomes dominant, resulting in a decrease in flow stress.
(2) Microstructural observations revealed that at lower deformation temperatures or higher strain rates, accelerated dislocation multiplication and accumulation lead to an increased content of substructures. Furthermore, the consistently high proportion of low-angle grain boundaries during warm compression indicates that dynamic recovery is the predominant softening mechanism for this alloy under warm deformation conditions.
(3) The Hensel–Spittel–Garofalo (HSG) was developed, along with an LSTM model optimized by the Harris Hawks Optimization algorithm. The HSG model achieved a relatively high *R* (0.9762) and a relatively low *AARE* (4.15%) between predicted and experimental flow stresses. The neural network model demonstrated superior performance with a high *R* (0.997) and a low *AARE* (3.98%). These results confirm that both models possess accurate predictive capabilities for the warm deformation behavior of the AA7075, with the HHO-LSTM model demonstrating superior predictive performance among them.

This study, while providing substantial insights, has numerous limitations. The HSG constitutive model and machine-learning models were developed and validated exclusively using isothermal hot-pressing test data. Their predictive accuracy for intricate non-isothermal industrial forming processes necessitates additional validation. Secondly, while the HHO-LSTM model exhibited exceptional predictive accuracy, its “black-box” characteristic restricts direct physical interpretation. Creating hybrid models that combine physical principles with data-driven techniques offers potential for attaining both high precision and interpretability.

## Figures and Tables

**Table 1:** The yield stress under various deformation situations.

*T* (°C)	$\dot{\varepsilon }$ (s^−1^)	$\sigma_{s}$ (Mpa)	*T* (°C)	$\dot{\varepsilon }$ (s^−1^)	$\sigma_{s}$(Mpa)	*T* (°C)	$\dot{\varepsilon }$ (s^−1^)	$\sigma_{s}$ (Mpa)	*T* (°C)	$\dot{\varepsilon }$ (s^−1^)	$\sigma_{s}$ (Mpa)
160	0.001	472.6	160	0.01	510.4	160	0.1	551.4	160	1	558.8
180	0.001	416.6	180	0.01	454.6	180	0.1	494.5	180	1	515.5
200	0.001	363.6	200	0.01	421.5	200	0.1	441.3	200	1	481.8
220	0.001	310.1	220	0.01	373.4	220	0.1	381.4	220	1	449.1

## Data Availability

The original contributions presented in this study are included in the article. Further inquiries can be directed to the corresponding authors.
